# Supplementary material for: Open-channel microfluidics via resonant wireless power transfer
Source: Nat Commun. 2022 Apr 6;13:1869. doi: 10.1038/s41467-022-29405-2 (PMC8987052; doi:10.1038/s41467-022-29405-2)
Supplement: Supplementary file 1 — Supplementary Information [file 41467_2022_29405_MOESM1_ESM.pdf]

Supplementary Material

Open-channel microfluidics via resonant wireless power transfer

Christopher T. Ertsgaard, Daehan Yoo, Peter R. Christenson, Daniel J. Klemme, Sang-Hyun Oh*

Department of Electrical and Computer Engineering, University of Minnesota, Minneapolis, Minnesota, 55455, U.S.A.

*Corresponding author. Email: sang@umn.edu

Electrode edge characterization

Characterization of the top electrode edge were measured after experiments by milling a cross-sectional slice using a focused ion beam (FIB) tool and a scanning electron microscope (SEM) (Helios NanoLab G4 dual-beam focused ion beam, FEI). Samples were first placed on a stage and tilted to an angle of 52°. A 10 µm × 10 µm region was milled across the edge of the top electrode that was 0.6 µm deep. The milled region was made near the drop reservoir location where microchannels had formed. This was followed by SEM images taken of the electrode's edge for sidewall height and angle, α, characterization. These values were then measured using ImageJ software. The sidewall height is important for determining the microchannel radius, R. Due to patterning a thick top electrode layer using lift-off (nominal height of 400 nm), sidewall deposition onto the photoresist created a non-uniformity of the electrode height at the edges after lift-off. We see this statistically as a bimodal distribution of the electrode height with regions either having or lacking the presence of sidewall deposition. Regions with sidewall deposition had a mean value of

1,453.9 nm ± 78.2 nm (n = 6) and regions without had a mean value of 535.6 nm ± 86.8 nm (n = 12). Since the appearance of sidewall deposition can vary between devices and even along the same edge of a single device, the total mean value across all 18 measurements was used for subsequent simulations. This resulted in a shift of the mean value of the electrode height to nearly double the nominal value with a large standard deviation (R = 842 nm ± 453 nm, n = 18) (Table S1). According to Equations 2 and 4, the microchannel radius influences threshold voltage for dielectric body-force actuation and has less influence within the conductive liquid regime. This explains the larger deviations in the threshold voltage for DI water whose onset depends on the electrode height/channel radius, R (Table S3). The averaged sidewall angle, α, measured was 71.8° ± 4.4° (n = 18) (Table S2)

Contact Angle Characterization

It has been shown that the contact angle of TiO_2 can be tuned using visible and UV exposure to light.[1] Devices were used within a week of the deposition of the 5 nm TiO_2 passivation layer were stored in a clean room environment wrapped in tin foil to protect against light-induced wettability and/or surface absorption of airborne particles that can affect the contact angle of the TiO_2 surface. Controlling the contact angle and preservation of the surface integrity was found to be significant factor in performance regarding actuation voltage and flow rate. Eight glass slides with the same stack of materials used for our device were used as test samples to characterize the contact angle of TiO_2 with an additional ten measurements from chips carrying multiple devices measured directly before use in experiments. The contact angle was measured using 1-2 drops of deionized water placed on different edges of the chip and a custom contact angle measurement setup. The sample was placed on a stage and a silver mirror (Thorlabs) directed the image of the drop profile through a long working distance objective microscope. The contact angle made with

the substrate was then measured on both sides of each drop's profile with ImageJ software and averaged. The average contact angle across all measured devices and test chips was 51.5° ± 9.2° (n = 18) (Table S2).

Capacitance Characterization of device

The device capacitance was characterized under both dry and wet conditions for determining its resonant circuit integration. When the device was dry, an LCR meter (4284A Precision LCR Meter, Hewlett Packard) was used to directly measure the capacitance. Before measurement, the LCR meter was calibrated by applying Short and Open Circuit conditions and 4 m cable capacitance calibration per standard operation. Measurements were made using a "Long" integration time, averaged over four measurements using a 100 kHz, 1 V_{RMS} measurement signal. The average capacitance measured was 1.21 nF ± 0.11 nF (n = 5) (Table S4). This value aided the choice in the resonant inductor used. Next, the resonant frequency found during resonant threshold voltage experiments (see Non-Resonant and Resonant Data acquisition section in the Supplementary Materials) was used to calculate the total capacitance of the device with solution present using the resonant frequency equation of a tank circuit, see Equation S1.

$$f_{RES} = \frac{1}{2\pi\sqrt{LC}} \quad , \tag{S1}$$

The average device capacitance with solution (for both DI and PBS solutions) calculated was 1.48 nF ± 0.67 nF (n = 4) (Table S4).

Modeling

All theoretical electric field and electric field gradient simulations for the different device geometries were modeled using a 2D electrostatic module in COMSOL Multiphysics. Custom

MATLAB scripts were written to process data and to generate theory curves and plots. When modeling the planar channel, two coplanar electrodes were separated by the specified gap width and filled with Al_2O_3 material ($\varepsilon_t = 9.1 \times \varepsilon_0$). A 20 nm Al_2O_3 passivation layer was also included for all planar gap geometries for more direct comparison with our stacked gap design that has a 20 nm Al_2O_3 layer covering the bottom electrode. The tangential electric field generated from the electrodes were averaged within semicircular regions defined directly above the gap and varied with a radius, R (Figure S3). Since the electric field generated from the gap is a fringe field, the magnitude is not equally distributed in space. Therefore, the magnitude was squared according to Equation 2 at each point and then averaged across the entire cross-sectional surface for each radius R. A MATLAB script found which channel radius and voltage resulted in an averaged electric field that met the surface tension threshold of Equations 2 for different gap widths. Literature values that specified their gap width (d), electrode width (w), and contact angle (θ) of their passivation layer were plotted and corrected to normalize the different contact angle substrates using our correction factor (see section: New Surface Tension Correction Factor, below). The literature data was plotted in Figure 1C, using a common contact angle of θ = 60°. The channel radius, R, was defined according to their provided equation for coplanar electrodes (Equation S2).

$$R = \frac{d}{2} + w \; , \tag{S2}$$

Our stacked gap structure was modeled in a similarly way. Electrodes were stacked and separated with a 20 nm Al_2O_3 film. The height of the top electrode was set to 900 nm and swept by one standard deviation of 453 nm as found in the Electrode edge characterization section above. This defined the radius of the microchannel, R. The substrate contact angle simulated was 55°. The sidewall angle, α, was then varied between 170°-40° (Figure 2B). The effective oxide thickness of

the 5 nm TiO_2 layer coated to normalize the contact angle compares to a ~0.6 nm layer of Al_2O_3. This sits on top of a 20 nm Al_2O_3 gap layer in which its combined effective thickness is within one standard deviation of the fabrication process (Table S1). Therefore, for simplicity it was left out of the simulation geometry. The electric field magnitude and threshold condition of the channel was calculated the same as above as a function of α. The dielectric constant, ε_L, and surface tension value, γ, used to model the liquids were $80 \times \varepsilon_0$ and 72 mN/m, respectively. It is important to note the practical limitation on the parameter space outlined in Figure 2C including dielectric breakdown (limiting operation below 7 V_{RMS} or 10 V amplitude, see Figure S2) and/or contact angle saturation (limiting sidewall angles less than 120°).

When modeling the dielectrophoretic rejection radius of polystyrene beads (see section Dielectrophoretic Filtering below) to explain the particle filtration of Figure 3H-I in the main text, a complex dielectric function was defined below (Equation S3).

$$\varepsilon^*(\omega) = \varepsilon(\omega) + i\frac{\sigma(\omega)}{\omega} , \tag{S3}$$

where σ is the conductivity of the material and is treated as a constant across the simulated RF frequencies, as is ε. The dielectric constant and conductivity values used for the polystyrene beads were $2.56 \times \varepsilon_0$ and 160×10^{-4} S/m, respectively. The conductivity of our solutions was modeled using our measured values.

Circuit modeling to generate the voltage gain curves seen in Figure 2C were performed using LTspice XVII software. A total of four different circuits were used experimentally (Figure S4). Non-resonant data consisted of an alternating current (AC) power source with an internal 50 Ω resistance in series with our capacitor-like device (using the mean capacitance of 1.5 nF as

characterized in section Capacitance Characterization of device (Figure S4A). Resonant data consisted of the previous configuration with an additional 100 μH inductor wired in series (Figure S4B). Wireless data consisted of two custom-made solenoid inductive antennas: the primary coil (L_p = 10 μH) for power transmission and the secondary coil (L_s = 50 μH) to receive power (Figure S4C). Finally, a smartphone device (Google Pixel 3a) was used to generate a Near Field Communication (NFC) input signal. The specific circuit hardware components of the smartphone device were unknown. However, using values readily available from vendors and assuming the phone drives with a digital 5-volt amplitude signal, the smartphone NFC circuit components were fit using an Inductor-Capacitor-Resistor model to the data (red components in Figure S4D). Values used for simulating the gain curves in Figure 2C of the main text are as follows: V_{IN} = 5-volt amplitude AC signal (13.5 MHz driving signal), R_1 = 50 Ω, L_1 = 2 μH, C_1 = 95 pF, k = 0.5, R_2 = 500 Ω. The motivation for the NFC theoretical curve in Figure 2C is less to demonstrate NFC circuit theory, but rather highlight the capability of our device to operate over broader frequencies (NFC carrier frequency) and with low-power mobile devices.

General Equation of the Threshold Condition for a Dielectric Body-force

It has been shown using the Korteweg-Helmholtz force density that tangential electric fields across the surface of a dielectric body (i.e. a phase or material boundary) can stimulate a localized pressure difference, Δp_D, across the drop and its environment, see Equation S4.

$$\Delta p_D = \frac{1}{2}(\varepsilon_L - \varepsilon_0){E_t}^2, \qquad \text{(S4)}$$

Assuming electrode ends are from the phase boundary of the dielectric such that the E-fields are completely orthogonal to the direction of fluid actuation, E_t is then the magnitude of these tangential E-field lines to the drop's surface, and ε_L and ε_0 are the dielectric constants of the liquid

and surrounding environment (air in this case), respectively. If E_t can be confined to a region to generate a Youngs-Laplace pressure with enough strength to overcome surface tension,[2,3] a confined fluidic channel of liquid will form, with a shape governed by the electrode geometry and corresponding E-field lines, assuming a capillary condition has not already been met. This pressure term is then inserted into the Gibbs Free energy equation (Equation 1 of the main text). For simplicity, temperature change dT are assumed to be negligible or lumped into a temperature dependent surface tension term, ($\gamma_i \rightarrow \gamma_i(T)$). The volume changes, dV, and surface area changes, dA_i, of the microchannel both carry the channel's length term, dz, which can be factored out. Rearranging terms the following condition is found, see Equation S5.

$$\Delta p_D * (S_c\, dz) \geq \; dz \textstyle\sum_i \gamma_i\, w_i \tag{S5}$$

Here, S_c is the cross-sectional area of the microchannel and w_i, is a contour length of that cross-section that is in contact with a material whose interface has a surface free energy density or surface tension γ_i. With the channel length, dz, canceling, the design problem becomes 2-dimensional, dependent on the geometry of the microchannel's cross-section. This can be used to reduce operating voltage through optimization of the electrode's design. To simplify Equation S5, the surface free energy of an interface, γ_i, can be written in terms of a more easily measurable Young's Contact angle, θ_i, if the surface tension of the liquid with the surrounding environment, γ, is known, see Equation S6.[37]

$$\gamma_i = \; -\gamma cos(\theta_i) \tag{S6}$$

Using this relation and replacing the dielectrophoretic pressure (Equation S4), we arrive at the following condition for channel flow, see Equation S7.

$$\frac{1}{2}(\varepsilon_L - \varepsilon_0){E_t}^2 \geq \frac{-\gamma}{S_c}\textstyle\sum_i \; w_i cos(\theta_i) \tag{S7}$$

Where ε_L and ε_0 are the dielectric permittivity of the liquid and surrounding medium, respectively. The electric field tangential to the drop's surface, E_t, that generates the sufficient pressure will, by definition be confined to the cross-sectional area of the channel S_c. A general condition on the electric field for liquid actuation of a microchannel with an arbitrary cross-section geometry is provided below (Equation S8).

$$E_t^{\,2} \geq \frac{-2\gamma \sum_i w_i \cos(\theta_i)}{S_c(\varepsilon_L - \varepsilon_0)} \tag{S8}$$

Equation S8 is essentially the surface area to volume ratio of the channel. This means, a characteristic length term will remain in the denominator such that the strength of E-fields will be inversely proportion to the characteristic dimension of the channel. This can either extremely help or hinder actuation depending on the sign on the surface energy in the numerator. If surface energy contained in the numerator is favorable for capillary, the sign will be negative and thus the inverse proportionality will rapidly reduce the E-field strength needed for actuation. Contrary, if the geometry is not favorable, the numerator will be positive and thus increasingly large E-fields will be necessary as the characteristic dimension of the channel is reduced. This can be a problem for open-channels in which a significant portion is exposed to a "hydrophobic" air/oil environment. The summation of Equation S8 will include the portion of the contour the liquid makes with its surrounding environment (e.g. Air → w_{air} , $\theta_{air} = \pi$) making the numerator positive unless a larger contour length can be made with the electrodes to reduce this energy cost.

Derivation of a Circular Sector Channel Body-force

For both the coplanar electrode design used in classic LDEP and for our stacked electrode design, a circular sector channel entrance is defined using a sector angle, α (defined in radians). The resulting cross-sectional area is provided below, see Equation S9.

$$S_c = \frac{1}{2}\alpha R^2 \tag{S9}$$

Where, R, is the radius of the circular sector (Figure 1B). Using this model, the contour exposed to the environment—in this case air ($\theta_{air} = \pi$), has an arc length of αR and the two contours of the cross-section in contact with the electrodes have a combined length of $2R$. Treating both electrodes as having the same contact angle, θ, we can substitute these dimensions into Equation S8 and find the threshold condition for a microchannel with a circular sector cross-sectional geometry, see Equation S10.

$${E_t}^2 \geq \frac{-4\gamma(2Rcos(\theta)-\alpha R)}{\alpha R^2(\varepsilon_L-\varepsilon_0)} \tag{S10}$$

After simplifying, the derived equation used in the main text (Equation 2) can be found.

General Equation for the Threshold Voltage within the conductive liquid regime

When operating in the conductive regime (see Conductive to Dielectric Cross-over Frequency section in the Supplementary Materials), charges can accumulate and screen the electric fields generated by the electrodes (Figure S6) and thus reduce the dielectrophoretic pressure to zero. We present the derivation for a purely conductive liquid threshold condition using the same surface tension model outlined above. Starting with Equation S8**,** the E_t term is set to zero—assuming it is completely screened by mobile charges in the solution (Figure S6), and thus the following condition is found, see Equation S11

$$\sum_i w_i \, cos(\theta_i) \ \geq \ 0 \tag{S11}$$

This result is the generalized Cassie law and indicates the condition in which spontaneous capillary action will occur. [37] Using the physics of EWOD, the Lippmann equation[6] provides a relationship between a voltage applied to a deformable body with mobile charges and its contact angle that it forms with the subsequent electrodes, see Equation S12.

$$cos(\theta) = cos(\theta_0) + \frac{\varepsilon_t}{2t\gamma} V^2 \quad \text{(S12)}$$

With EWOD utilizing a passivation layer over the electrodes, the new effective contact angle θ that results after applying a potential V, depends on the initial Young's contact angle with the substrate, θ_0, and the permittivity of the dielectric passivation layer, ε_t, that separates the drop from the electrode with thickness, t. This equation assumes the mobile charges can instantly screen the electric fields (i.e. perfect conductivity) and ignores the effect of contact angle saturation in which the effective contact angle, θ, approaches some minimum, fixed value. Therefore, Equation S12 will be considered as an upper bound for conductive solutions and will be used to describe the initial onset of actuation such that saturation effects can be ignored. Therefore, if Equation S11 is not already satisfied, a voltage can be applied to tune the contact angle until it is satisfied and thus permits microchannel formation. The generalized form, which includes the Lippmann equation (Equation S12) can be written as follows:

$$\sum_i w_i \, cos(\theta_i) + \sum_k \frac{w_k \varepsilon_k}{2\gamma t_k} {V_k}^2 \geq 0 \quad \text{(S13)}$$

where i is summed over all contour interfaces while k is summed over only those interfaces which drop a voltage V_k. The thickness of the dielectric passivation layer, t_k, separates the mobile charges from reacting with the metal electrodes and has a dielectric constant of ε_k. This layer is the dielectric film in which EWOD (electrowetting on dielectric) gets its name. Assuming for simplicity the magnitude of potential dropped over the k interfaces are the same (i.e. $V_k = V$), a generalized critical voltage relation can be solved for a purely conductive solution, see Equation S14.

$$V^2 \geq \frac{-2\gamma \sum_i w_i \, cos(\theta_i)}{\sum_k \frac{w_k \varepsilon_k}{t_k}} \quad \text{(S14)}$$

If we use the same circular sector channel as before (Figure 1B and S6), Equation S14 will simplify to Equation 4 provided in the main text.

New Surface Tension Correction Factor

Due to literature LDEP experiments with different reported contact angle substrates (θ_R), we define a means to map their reported threshold voltage (V_R) to an equivalent threshold voltage (V_E) on a substrate with contact angle (θ_E). Since LDEP experiments use coplanar electrode arrangements ($\alpha = \pi$), we can use Equation 2 of the main text to define a simple multiplicative correction factor, see Equation S15

$$V_E \approx \frac{\pi - 2cos(\theta_E)}{\pi - 2cos(\theta_R)} V_R \tag{S15}$$

Equation S15 is used to normalize literature data with varying contact angles to a common substrate with the same contact angle for more direct comparison with our theory and experimental data used in Figure 1C. It is important to note, this normalization does not appreciate the effects of the passivation layers that were used used. These layers will consume a portion of the electric field used for actuation and thus reduce efficiency the thicker they become. This of course will increase the operating voltage and have a more notable affect as the electrode gaps are reduced due to more of the confined fringe fields buried within the passivation layers. This is believed to be a contributing factor as to why the threshold voltage for the 2 μm wide gap literature data[9] (which specifically contained an oxide and nitride passivation layer that was 0.6 μm thick) were larger than our theoretical prediction for 1 μm wide gaps after our correction.

Conductive to Dielectric Cross-over Frequency

Depending on the frequency of the driving signal, either displacement or Ohmic currents will dominate the system in which either Equation 2 or 4 in the main text will determine the dominate mode of actuation. This frequency response relates to the charge relaxation time of the ionic species in solution. Therefore, the crossover frequency between the conductive to dielectric regime depend is found when the displacement current and Ohmic terms are set equal and will depend on the conductivity of the solution, electrode geometry and equivalent circuit model.[6] A simple estimate can be made by considering just the liquid's bulk electrical properties, see Equation S16.

$$\omega_c = \frac{\sigma_L}{\varepsilon_L} \tag{S16}$$

Where σ_L is the conductivity and ε_L is the dielectric permittivity constant of the liquid, respectively. As ionic material is added to the solution, the conductivity of the solution will increase and result in larger frequencies needed to operate in the displacement current dominate regime (i.e. Equation 2 of the main text). Thus, conductive solutions are problematic for classic LDEP which depends on dielectric body-forces for actuation. As an example, water ($\varepsilon_L = 80$) with dissolved salt ions that has a conductivity of 1 S/m (typical physiological conditions) has a crossover frequency around 100 MHz which is challenging when operating at 50-100 V_{RMS} for classic LDEP that uses coplanar electrodes.

Flow-rate estimations

When considering flow-rates and fluid velocity for microfluidic channels that actuate orthogonal to gravity, the classic capillary "rise-to-height" derivations do not apply. Rather friction or fluid drag is the dominate factor opposing fluid velocity. Furthermore, at this scale inertial fluid

components become negligible (i.e. a low Reynold's number regime). Therefore, the condition when the actuation force, F_z, balances the drag force, F_{drag}, must be found,[37] see Equation S17

$$\frac{d(mu)}{dt} = F_z - F_{drag} \approx 0 \tag{S17}$$

Here, m and u are the channel's mass and velocity, respectively, and comprise the channel's inertial terms which are near zero at this scale. The actuation force, F_z, can be found by taking the gradient of the Gibb's free energy (Equation 1 of the main text), specifically along the z-direction—defined as the direction of flow and opposite of the gradient, see Equation S18.

$$F_z = -\nabla_z G = \gamma \sum_i w_i \, cos(\theta_i) + \sum_k \frac{w_k \varepsilon_k}{2t_k} {V_k}^2 + \Delta p_D S_c \tag{S18}$$

Here both actuation mechanisms are included where dielectric/displacement current dominate conditions will result in the dielectric pressure term, Δp_D, (Equation S4) dominating liquid actuation and thus a negligible potential, V_k, will be dropped over a given single contour. Conversely, if the solution is purely conductive such that the majority of the input voltage drops over the dielectric passivation layer, the dielectrophoretic pressure term, Δp_D, will be zero and electrowetting will dominate actuation.

To solve the drag force equation, the channel shear stress, τ, must be known with an exact description of the fluid velocity profile, u, using the Navier-Stokes equation. Contrary to classic microfluidics or pipe flow where the fluid velocity is maximal at the center of the channel, (i.e. $R = 0$), the open-channel should have maximal fluid velocity existing at the open-phase boundary exposed to the ambient air environment which poses negligible shear stress. While a complete description of the flow profile is beyond the scope of this work, an approximation for the drag force can be provided to appreciate the general evolution of the channel over time as it follows a square-root function in time. By defining the shear stress in terms of an averaged Fanning friction factor, f, the drag force can be written as a function of the channel length, $z(t)$, see Equation S19.

$$F_{drag} = \mu\, f \,\mathrm{Re}\, \frac{d}{dt} z(t)^2 \tag{S19}$$

The drag force follows a squared function of the channel length, with the fluid's dynamic viscosity, μ, a fanning friction factor, f, and the Reynolds number, Re, scaling its strength. The Fanning friction factor can be written in terms of the mean shear stress of the open-fluidic channel, τ, and the mean fluidic velocity profile, u, i.e. dz/dt, see Equation S20.

$$f = \frac{2\,\bar{\tau}}{\rho \bar{u}^2} \tag{S20}$$

Here, ρ, is the mass density of the fluid. While the product, $f\,Re$, contains a velocity term making the equation for $z(t)$ a high-order differential equation, an approximation can be made to assume that this product is constant over the time window of liquid actuation. Additionally, the factor $z(t)^2$ can be treated as a lumped variable as was the approach of others.[37, 4] In doing so, $z(t)$, can be approximated as a root function with time using Equation S17, see Equation S21.

$$z(t) = \sqrt{\frac{F_z}{\mu\, f\, \mathrm{Re}}\, t} \tag{S21}$$

Here, F_z, is the actuation force (Equation S18) and the friction factor, f, can be empirically fit and was found to be in good agreement with our open-channel evolution data. The PBS spiral channel length, $z(t)$, of Figure 3A-C was observed under fluorescent microscopy, in which the channel length was recorded every 5 s after a continuous 3.5 V_{RMS} supply voltage was applied. Averaging over the 4.5 minutes of channel formation (30 s baseline was recorded with zero input voltage at the beginning), a mean fluid velocity of 1.69 µm/s was found. The channel length, $z(t)$, followed a square-root function with time that was then fit to Equation S21 using a non-linear least squares (NLS) approximation with good agreement (RMS error of 21.7 µm), see Figure S7. The NLS was implemented using a MATLAB built-in NLS function in which the mean device parameters (Tables S1 – S2) were used to defined the actuating force, F_z, (Equation S18**)** under the purely

conductive wetting regime (i.e. $\Delta p_D = 0$ for PBS solution) with a 20 nm alumina oxide layer (t = 20 nm, $\varepsilon_t = 9.1 \times \varepsilon_0$) and an input voltage of 3.5 V_{RMS}. The room temperature (25 C) values for water were used to define the dynamic viscosity ($\mu = 8.9 \times 10^{-4}$ [Pa*s]), surface tension ($\gamma = 72 \times 10^{-3}$ [N/m]), and mass density ($\rho = 997$ [kg/m^3]) terms. A Reynolds number of $Re = 1.6 \times 10^{-6}$ was approximated using the mean fluid velocity. Finally, the NLS found an "effective" mean shear stress of the open-fluidic channel, τ_{eff}, to be 42.4 Pa (used to define the Fanning friction factor of Equation S20) to provide the best-fit data in Figure S7.

The average channel widths ranged between 1-5 µm as the channel was extruded. Assuming the circular sector geometry, this would equate to a flow rate of 0.3-1 pL/min per channel which was 0.2% of the drop's volume per minute per channel averaged over the five minutes. With a general estimation of the fluid velocity, flow rate, and channel progression as a function of time provided, more extensive study and optimization is reserved for future work with the purpose of this section to provide an appreciation of the fluid dynamics trend and approximated flow rates.

Clausius-Mossotti Factor

The sign of the real part of the Clausius–Mossotti Factor (CMF) determines the direction particles are guided (toward or away) as they approach the microchannel entrance[42] (Equation S22):

$$f_{CMF}^{*} = \frac{\varepsilon_P^{*}(\omega) - \varepsilon_L^{*}(\omega)}{\varepsilon_P^{*}(\omega) + 2\varepsilon_L^{*}(\omega)} \tag{S22}$$

Where ω is the angular frequency of the driving voltage signal and ε_P^* is the complex dielectric function of the particle and ε_L^* is the complex dielectric function of the liquid medium immersing the particle, respectively. When the particles are more polarizable than the liquid solution, the real

part of the CMF will be positive and result in particles trapped towards the substrate surface before entering the microchannel. Conversely, if the liquid medium is more polarizable the CMF will be negative resulting in a repelling force on the particles away from the entrance of the channel (Figure S8A).

Dielectrophoretic Filtering

The dielectrophoretic force exerted on a spherical particle depends on the size of the particle, gradient of the electric field generated by the electrodes, and the real part of the Clausius-Mossotti factor (CMF) described in the preceding section. As particles in solution wander or flow to the entrance of the microchannel, their thermal energy will determine whether they can breach the dielectrophoretic gate or be rejected from entering the microchannel. The 1D thermal force (expressed as Brownian motion) on a spherical particle depends on the particle's size and temperature as shown below (Equation S23).

$$F_{th} = \frac{k_B T}{2r_P} \qquad \text{(S23)}$$

Where F_{th} is a thermally derived force contributing to Brownian motion, k_B is the Boltzmann constant, T is the ambient temperature and r_P is the particle's effective radius. This force must be greater than the dielectrophoretic radial force (perpendicular to the microchannel) (Equation S24) for the particle to enter the microchannel.[42]

$$F_{sphere} = \pi\, \varepsilon_L\, {r_P}^3\, Re\{f_{CMF}^*\}\, \nabla|E|^2 \qquad \text{(S24)}$$

Here ε_L is the dielectric constant of the liquid medium, and the ${f_{CMF}}^*$ is calculated as prescribed in Equation S22. The $\nabla|E|$ term is the gradient of the electric field generated from the nanogap. The cubic dependence of the dielectrophoretic force on the particle radius means that large particles are rejected much more strongly than small particles. This can be exploited as a filtration

mechanism, where large particles and impurities are rejected from entering the liquid channel at the entrance (Figure 3H-I).

The size of particles, r_p, permitted to enter depends on the microchannel radius, R, and operating voltage used. From simulations, the gradient of the electric field is strongest near the surface of the substrate/electrode (Figure 1D) and penetrates further as the operating voltage is increased. For a given voltage used to extrude the liquid, the radial DEP force (Equation S24) can interact with particles of a certain size at a unique distance within the microchannel (when comparing particles with identical ε_P*). This distance where the DEP force can trap/repel oncoming particles we define as a "gated" radius, $G_R(V, r_p)$. If this gated radius extends beyond the microchannel radius, R, then particles of size, r_p, are rejected from entering (Figure S8D), otherwise an annulus is formed in which particles are free to enter (Figure S8B). Theoretical plots of this gated radius were simulated using COMSOL for polystyrene beads of various sizes as a function of operating voltage (Figure S8C). This was performed for both DI water and PBS solution, in which particles are trapped rather than repelled at the gate radius, respectively. When using our digital logic voltage level of 3.5 V_{RMS}, polystyrene beads larger than 15 nm are rejected from a microchannel with our nominal radius of 400 nm. When using the mean channel radius of ~900 nm, polystyrene beads larger than 50 nm are rejected (Figure S8C). The 190 nm particles have a rejection radius greater than 4 µm and thus are continually rejected from entering the microchannel as observed experimentally (Figure 3H-I).

Device Parameter	Symbol	Number of measurements	Nominal Value (nm)	Mean (nm)	Standard Deviation (nm)
Top Electrode Height	R	18	400	842	453
Gap Spacing	g , t	3	20	20.1	0.6
Passivation Layer	-	7	5	4.7	0.2

Table S1. Tabulated fabrication dimensions. The corresponding symbols used within the text are included for reference and a sample standard deviation is used in the last column. The average electrode height is double the nominal value with a large standard deviation. This is due to regions of the electrode edge where sidewall deposition remained after the lift-off process. If we compare statistics of measured regions without this sidewall deposition to regions with sidewall deposition, we find a bimodal distribution. The mean electrode height for regions without a sidewall deposition was 535.6 nm ± 86.8 nm (n = 12) and with a sidewall deposition 1,453.9 nm ± 78.2 nm (n = 6). The large variability of the electrode height between samples and even along the same edge of a single sample, creates variability in the microchannel radius, R, in which dielectric body-force operation is sensitive to. In general, larger R requires less surface energy and thus can reduce operating voltage, Equation 2 of the main text.

Device Parameter	Symbol	Number of measurements	Mean (deg.)	Standard Deviation (deg.)
Contact Angle	θ_0	18	51.5°	9.2°
Sector Angle	α	18	71.8°	4.4°

Table S2. The measured static contact and sector angle are tabulated for a Concuss-Finn assessment. The corresponding symbols used within the text are included for reference and a sample standard deviation is used in the last column.

Circuit Configuration	Number of devices tested	Mean (V_{RMS})	Standard Deviation (V_{RMS})
Threshold Voltage (DI Water)	9	4.53	0.71
Resonant Threshold (DI Water)	4*	1.41	0.14
Res. Wireless Threshold (DI Water)	7	1.57	0.34
Threshold Voltage (1×PBS)	6	1.70	0.46
Resonant Threshold (1×PBS)	4*	0.53	0.12
Res. Wireless Threshold (1×PBS)	6	0.95	0.74

Table S3. The measured threshold voltages are tabulated for reference. Statistics are included for six conditions in which the threshold voltage was recorded: Non-Res. DI water, Res. with wired connection DI, Res. Wireless DI water, Non-Res. 1×PBS, Res. with wired connection 1×PBS and Res. Wireless 1×PBS. The sample standard deviations are used in the last column. Starred samples were used to collect data simultaneously for the Non-Res. and Wired Res. data points where the input voltage and actual voltage dropped across the device could be recorded.

**Device Characterization**	**Number of devices tested**	**Mean (nF)**	**Standard Deviation (nF)**
Line electrode capacitance (Dry)	5	1.21	0.11
Line electrode capacitance (Wet)	4	1.48	0.67

Table S4. The measured and calculated device capacitance are tabulated for reference. The device capacitance was measured when the samples were dry before operation. Due to the possibility of stimulating microchannel formation with the measurement signal, the resonant frequencies used from the Resonant threshold voltage experiments were used to calculate the capacitance of the device with solution present using Equation S1. This was averaged for both the DI and PBS solution measurements.

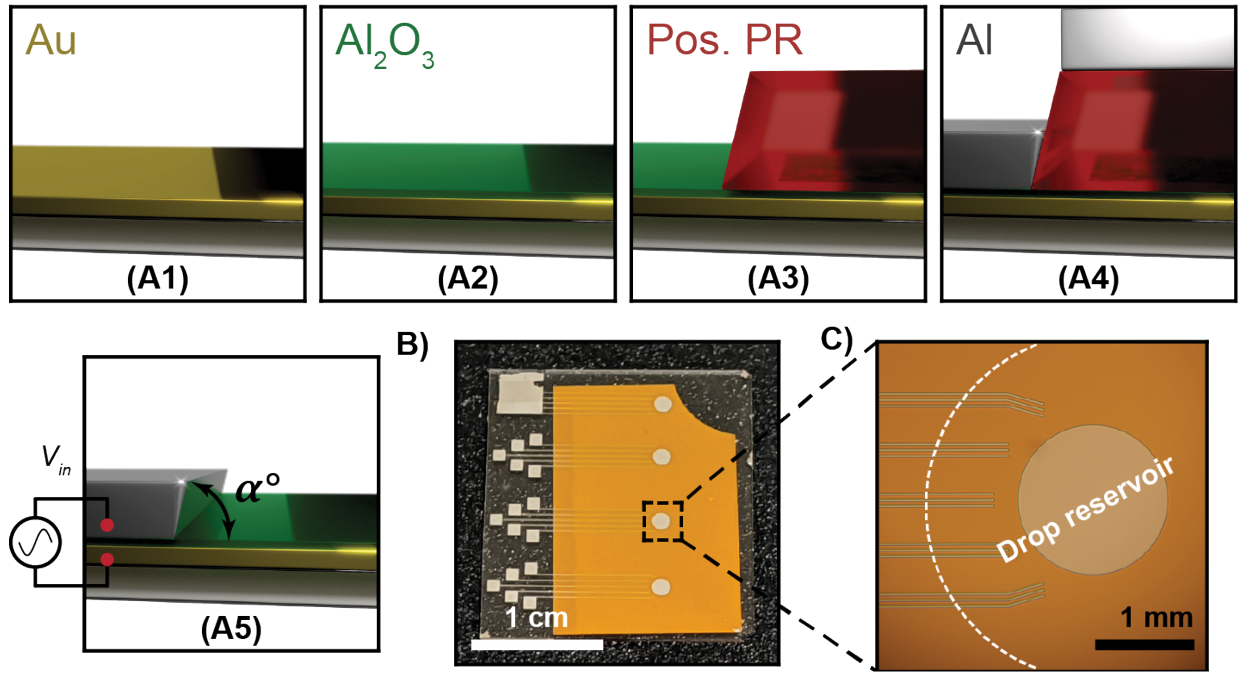

Fig. S1. Fabrication and electrical characterization of our stacked design. (**A**) The fabrication steps: (**Step 1**), First, the bottom gold electrode is patterned on glass. (**Step 2**) Second, a 20 nm Alumina film is grown using atomic layer deposition (ALD). (**Step 3**) Next, a positive photoresist is used with an obtuse angle sidewall angle to pattern the top electrode. (**Step 4**) This is followed by aluminum deposition (400 nm) through evaporation. (**Step 5**) Finally, lift-off defines a top electrode with an acute sidewall angle, α, and bias is applied across the ALD layer. The 5 nm TiO_2 passivation layer is not shown. (**B**) An image of one complete chip. Electrical contact is made to the top electrode on the glass region of the device. (**C**) A microscope image of the region where the drop is placed. The circular drop placement pad is patterned to guide eye so that the placement of the drop overlaps the top electrodes, designed here as a set of multiplexed lines.

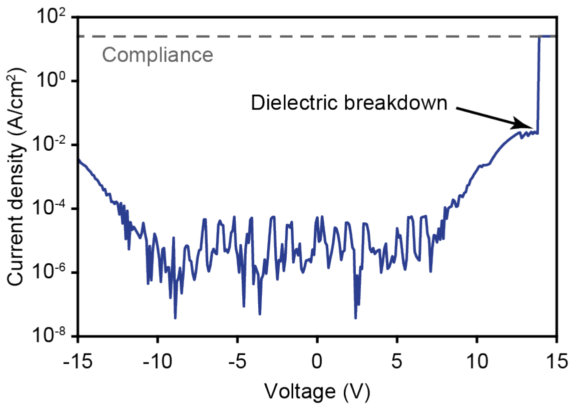

Fig. S2. Device IV Breakdown. An I-V curve to characterize when electrical breakdown occurs for our 20 nm Al_2O_3 gap. The data shows the gap is shorted after applying ~12 volts DC. The asymmetry in the I-V curve is believed to be due to the asymmetric choice in electrode materials (one is gold the other aluminum) which can affect the density of defects/trap sites at their respective interfaces with the dielectric gap. Regardless of asymmetry or polarity, since we apply an AC signal across the gap, it is important that a 10-volt amplitude signal is not used to prevent device failure. Source data is provided as a Source Data file.

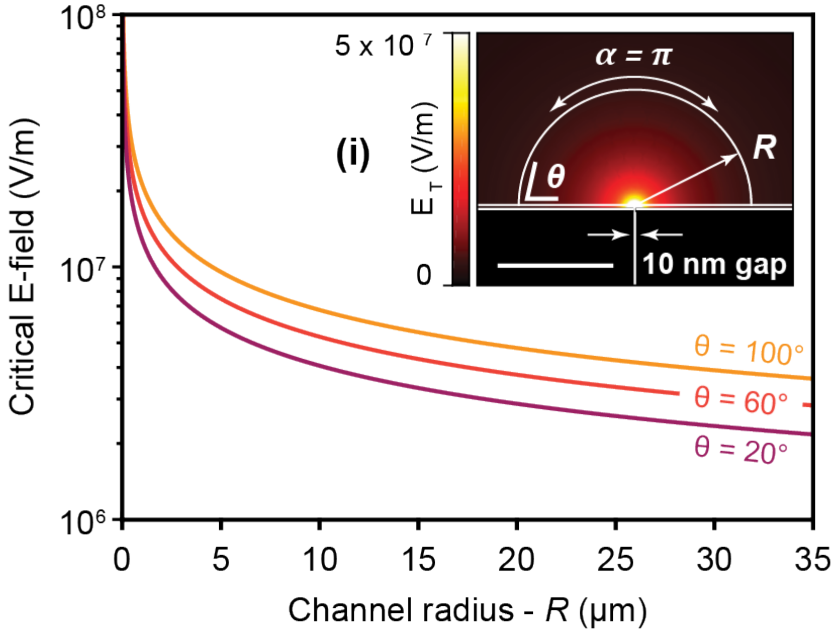

Fig. S3. Critical electric field for dielectric body-force actuation using coplanar electrodes. Simulations of the electric field distribution for coplanar electrodes were used to determine the threshold voltages in Figure 1C of the main text. Due to the inverse relation on surface tension to channel radius, the critical electric field (Equation 2) grows rapidly as tighter confinement of the liquid is attempted. The critical electric field extracted from the model is shown plotted against channel radius, and the effect of substrate contact angle can also be seen by comparing the curves. In general, larger electric fields are needed for larger contact angle (or more hydrophobic) surfaces. **(i)** Inset: field distribution map of coplanar electrodes for a 10 nm alumina gap after applying 10 V. Scale bar is 1 μm. The tangential electric field is squared, then averaged within the semicircular cross-sectional area (outlined here with a radius of 1 μm). Source data is provided as a Source Data file.

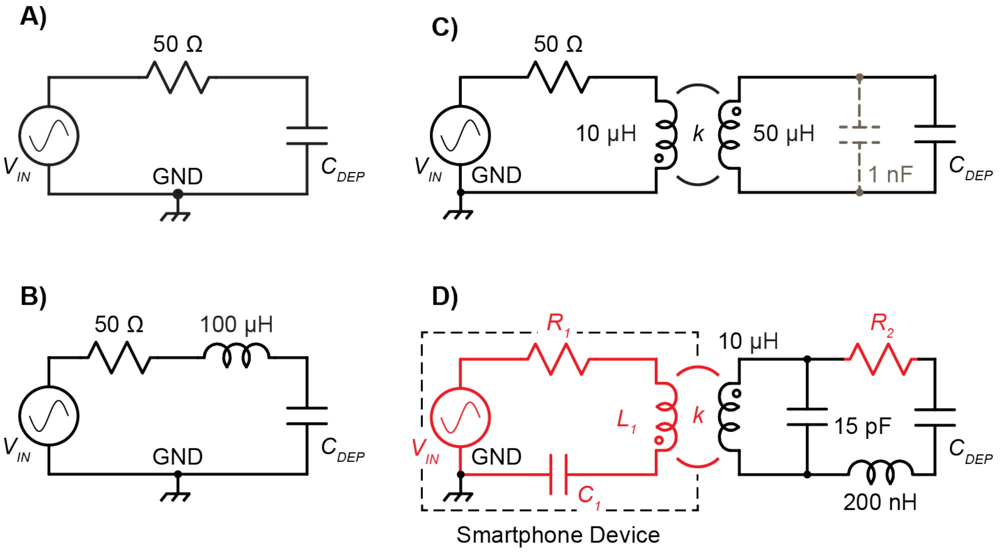

Fig. S4. Circuits used for experimentation and modeling (A) An RC circuit used for non-resonant threshold voltage experiments and simulation. As measured from our linear electrode geometry, the value simulated for our device capacitance, C_{DEP}, was 1.5 nF. The 50-ohm internal resistance of the function generator was also included in our simulation that powered the circuit with voltage V_{IN}. **(B)** An LCR circuit used for resonant threshold voltage experiments in which C_{DEP} and the internal resistance were the same as in "A." **(C)** A wireless inductive coupling circuit with a coupling coefficient k. This circuit was used for resonant wireless actuation and the C_{DEP}, and internal resistance were taken to be the same as "A" and "B". For PBS solutions, experimentally operating conditions were more sensitive to deviations in the device capacitance. A 1 nF parallel capacitor was used to make operation more robust while still dropping the same threshold voltage over both the device and capacitor. **(D)** Diagram of the Near Field Communication (NFC) circuit that was both simulated and tested experimentally. The circuit on the left was enclosed by the smartphone device and modeled as a simple LCR series circuit. The electric values of the components in red were fit to our experimental data using values readily available from vendors. The voltage drop across the device and NFC frequency were monitored using an oscilloscope.

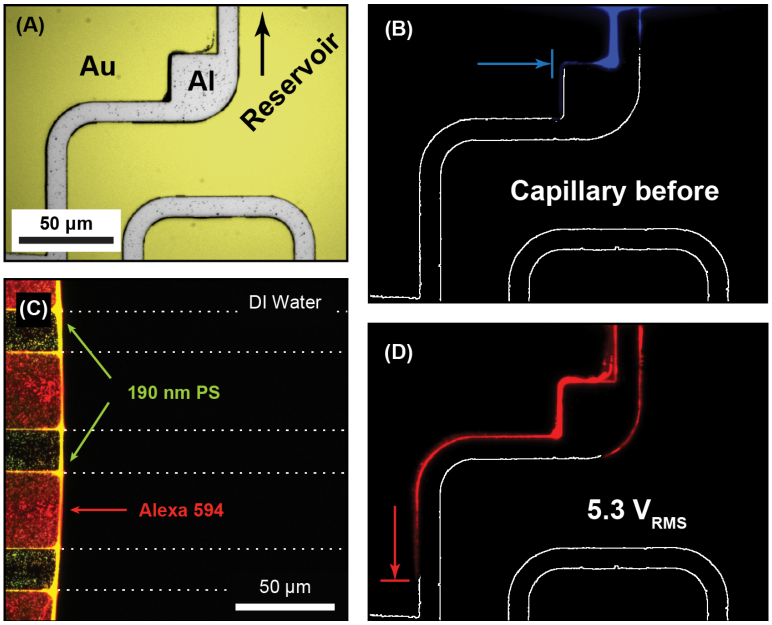

Fig. S5. Flow functionality of DI water (A) Top-down, bright-field image of a spiral aluminum top electrode device with a DI water reservoir drop positioned directly out of view. The Au bottom electrode (colored yellow) is covered with a 20 nm Alumina film to electrically separate from the top electrode. **(B)** Fluorescent image of Alexa dye molecules in the DI microchannel. Initially, capillary action pulls a small microchannel before voltage is applied (colored blue). The edges of the spiral electrode in white are added to outline the microchannel path the liquid will follow. **(C)** A particle filtration experiment with DI water. Due to positive dielectrophoresis existing at the regions where channel formation is to occur, large conglomerates of PS beads can be seen trapped and impeding flow. The frequency or solution conductivity must be addressed to operate in the negative dielectrophoresis regime so that particles are repelled from the microchannel entrance and channel formation can occur. **(D)** Using the same device in Panels "A" and "B" arbitrary flow pattern is demonstrated for DI water. In this case, a 5.3 V_{RMS} signal is applied (larger than the PBS solution due to meeting a higher threshold condition) using the Non-resonant circuit. Upon doing so, the channel grows in length compared to panel "B" and maneuvers around sharp angles and along curved paths.

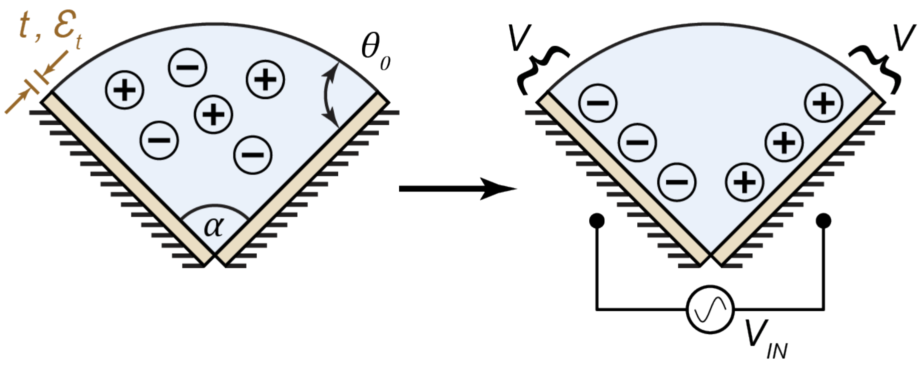

Fig. S6. Schematic of a circular sector channel geometry for a conductive liquid. A circular cross-section geometry configuration for liquids with mobile conductive charge. The channel defines the geometry used to define Equation 4 of the main text (similar to Figure 1B). A dielectric film with thickness, t, and dielectric permittivity, ε_t, passivates two electrodes that compose the legs of the circular sector with sector angle, α. The initial contact angle without electrical bias is θ_0. After applying a voltage across the two electrodes, mobile charges in the solution move to screen the electric fields. If the solution is treated as a perfect conductor, the electric fields will be terminated across the electrode and its respective "sheet" of charges. The simple Lippmann equation (Equation S12) makes these assumptions and treats all the voltage dropping across the dielectric film layer. While this is a simple demonstration of the phenomenon that occurs and ignores the effects of the double-layer, frequency dependence, etc., it can be used to gain a theoretical bound for actuation of conductive liquids.

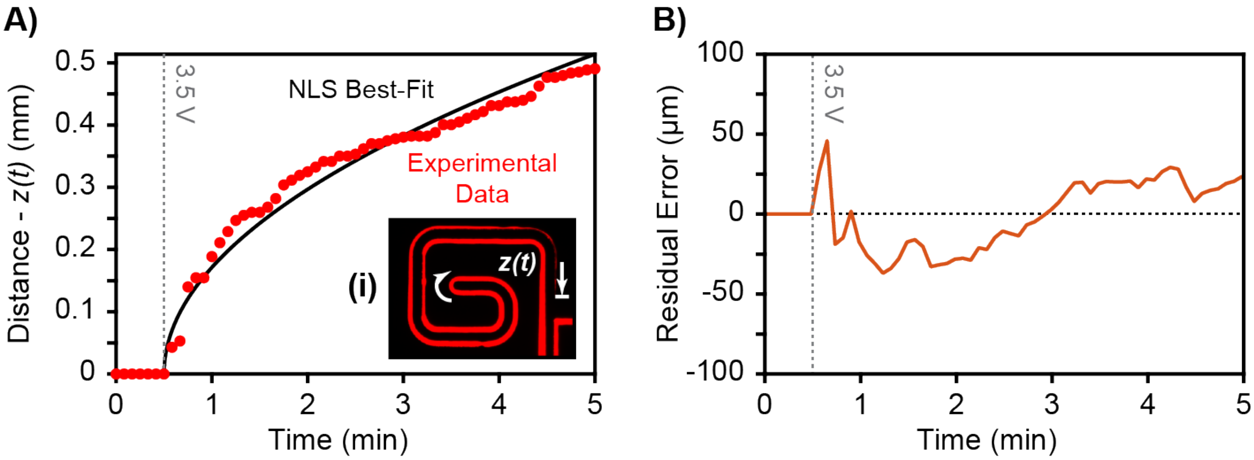

Fig. S7. Channel Flow dynamics. (**A**) The channel length, *z(t)*, of Figure 3A-C was recorded in time after a 3.5 V_{RMS} signal was applied after 30 s. This data was fit to Equation S21 using a non-linear least squares approximation. The average fluid velocity over five minutes was 1.69 µm/s which resulted in an estimated Reynold's number of $Re = 1.6 \times 10^{-6}$ and a fitted "effective" shear stress of the OMEF channel of $\tau_{eff} = 42.4$ Pa. The average fluidic channel widths ranged between 1-5 µm resulting in an estimated flow rate of 0.3-1 pL/min per channel. This equates to 0.2% of the drops volume per channel per minute. Source data is provided as a Source Data file. (**B**) A plot of the residual error between the best-fit function and the corresponding empirical data. An RMS error of 21.7 µm was found.

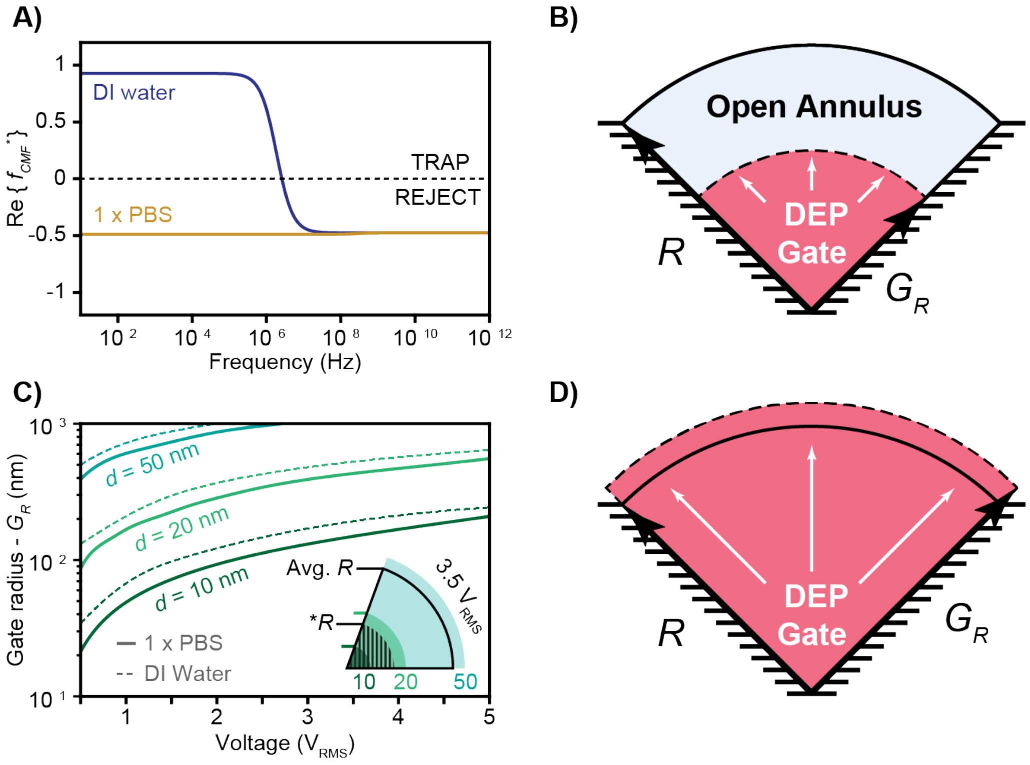

Fig. S8. Demonstration of particle filtration using dielectrophoresis. (**A**) The real part of the Clausius-Mossotti factor (CMF) as a function of frequency for polystyrene (PS) in DI water and PBS buffer. With our operating frequency of 100 kHz, PS beads are trapped in DI water and repelled in PBS, preventing their entrance into the liquid channel. (**B**) Schematic of the microchannel cross-section, showing a condition in which particles can enter. If the voltage used for liquid actuation results in a gated radius, G_R, (red area) that is less than the microchannel radius, *R*, then particles can enter through the "Open Annulus" above (light blue) and thus be transported down the microchannel. (**C**) The theoretical gated radius, G_R, for PS beads as a function of input voltage of various diameters. **Inset**: A visual aid comparing each gate radii (green) to the size of the channel entrance outlined in black (average *R* and nominal—$^{*}R$). (**D**). Similar schematic as "B," in which the gated radius (red area) is larger than the microchannel radius and thus particles cannot enter or be transported down the microchannel. Source data is provided as a Source Data file for Figures S8a and S8c.

Supplementary Materials References

1 Stevens, N., Priest, C. I., Sedev, R. & Ralston, J. Wettability of photoresponsive titanium dioxide surfaces. *Langmuir* **19**, 3272-3275, doi:10.1021/la020660c (2003).

2 Degennes, P. G. Wetting - Statics and dynamics. *Reviews of Modern Physics* **57**, 827-863, doi:10.1103/RevModPhys.57.827 (1985).

3 Liu, H. & Cao, G. Effectiveness of the Young-Laplace equation at nanoscale. *Scientific Reports* **6**, 23936, doi:10.1038/srep23936 (2016).

4 Bosanquet, C. H. On the flow of liquids into capillary tubes. *Philosophical Magazine* **45**, 525-531, doi:10.1080/14786442308634144 (1923).
