# Supplementary material for: Open-channel microfluidics via resonant wireless power transfer
Source: Nat Commun. 2022 Apr 6;13:1869. doi: 10.1038/s41467-022-29405-2 (PMC8987052; doi:10.1038/s41467-022-29405-2)
Supplement: Supplementary file 3 — Description of Additional Supplementary Files [file 41467_2022_29405_MOESM3_ESM.pdf]

**Description of Additional Supplementary Files**

**File Name: Supplementary Movie 1**

**Description:** Using the device shown in Figure 3a, a spiral fluid path is demonstrated resulting in a total channel length > 1mm from the reservoir drop. When the drop was first placed, spontaneous capillary flow was observed due to a sidewall exceeding the capillary limit. The liquid channel traveled ~684 µm before terminating at the center of the spiral before the movie begins. The first 30 s of the movie show the channel before a voltage bias is applied to serve as a baseline of the channel's position. After 30 s, a 3.5 $V_{RMS}$ voltage is applied resulting in the liquid channel wrapping around the outer edge of the top electrode. It then travels an additional ~566 µm in 5 minutes. The total length of the channel was ~1.25 mm from the drop and 5 µm in width. The real-time clock is included in the top corner of the movie and the application of the 3.5 $V_{RMS}$ is indicated at the time it is applied.

**File Name: Supplementary Movie 2**

**Description:** Using a device like that of Figure 3d, progression of ~25 parallel fluidic channels (as the channels from each of the 50 edges merged with their nearest neighbor) carrying FITC-labeled norovirus-like-particles is demonstrated. No voltage bias is applied during the first minute followed by five minutes of 3.5 $V_{RMS}$ voltage bias. The channels increase in length and intensity as the viral capsids are extruded out of the reservoir drop. Infrared absorption spectroscopy of the channel region was performed confirming the presence of viral proteins within the channels (Figure 3g). The real-time clock is included in the top corner of the movie and the application of the 3.5 $V_{RMS}$ is indicated at the time it is applied. The fluorescence is artificially colored purple in this video to visually separate experiments from those of the Alexa Fluor-594 molecules.

**File Name: Supplementary Movie 3**

**Description:** Using a device like that of Figure 3d, two reservoirs are mixed using a wired RC circuit. The left reservoir (green) contained green fluorescent protein (GFP) and the right reservoir (red) contained Alexa Fluor-594. After 30 s, a 4.2 $V_{RMS}$ signal was applied and solution from each reservoir was pulled into the ~25 channels. Conglomerates of GFP were extruded from the GFP reservoir into individual channels and were labeled red by the Alexa dye after mixing using amine chemistry. Images were taken by toggling between two filter cubes and overlaying their images at each time stamp. The real-time clock is included in the top corner of the movie and the application of the 4.2 $V_{RMS}$ is indicated at the time it is applied.

**File Name: Supplementary Movie 4**

**Description:** Using a device like that of Figure 3d, two reservoirs are mixed using a wireless LCR circuit. The left reservoir (green) contained green fluorescent protein (GFP) and the right reservoir (red) Alexa Fluor-594. After 60 s, a 3.5 $V_{RMS}$ signal was applied and solution from each reservoir was pulled into the ~25 channels. Images were taken by toggling between two filter cubes and overlaying their images at each time stamp. The real-time clock is included in the top corner of the movie and the application of the 3.5 $V_{RMS}$ is indicated at the time it is applied.

**File Name: Supplementary Movie 5**

**Description:** Using a device like that of Figure 3a, a spiral fluid path is extruded of Alexa Fluor-594 molecules upon applying a wireless near field communication (NFC) signal emitted by a smartphone. A thin thread of spontaneous capillary can be seen before voltage bias within the first 30 s due to capillary action. After bringing the smartphone device < 1 centimeter from the device antenna, wireless power is transferred and used to extrude the sample solution. The length and intensity of the channel can be seen to increase during smartphone NFC application. The real-time clock is included in the top corner of the movie and the application of the smartphone is indicated at the time it is applied. The fluorescence is artificially colored cyan in this video to visually separate the smartphone experiment from the other Alexa Fluor-594 molecule based experiments.
