# Supplementary figures and images for: Open-channel microfluidics via resonant wireless power transfer
Source: Nat Commun. 2022 Apr 6;13:1869. doi: 10.1038/s41467-022-29405-2 (PMC8987052; doi:10.1038/s41467-022-29405-2)

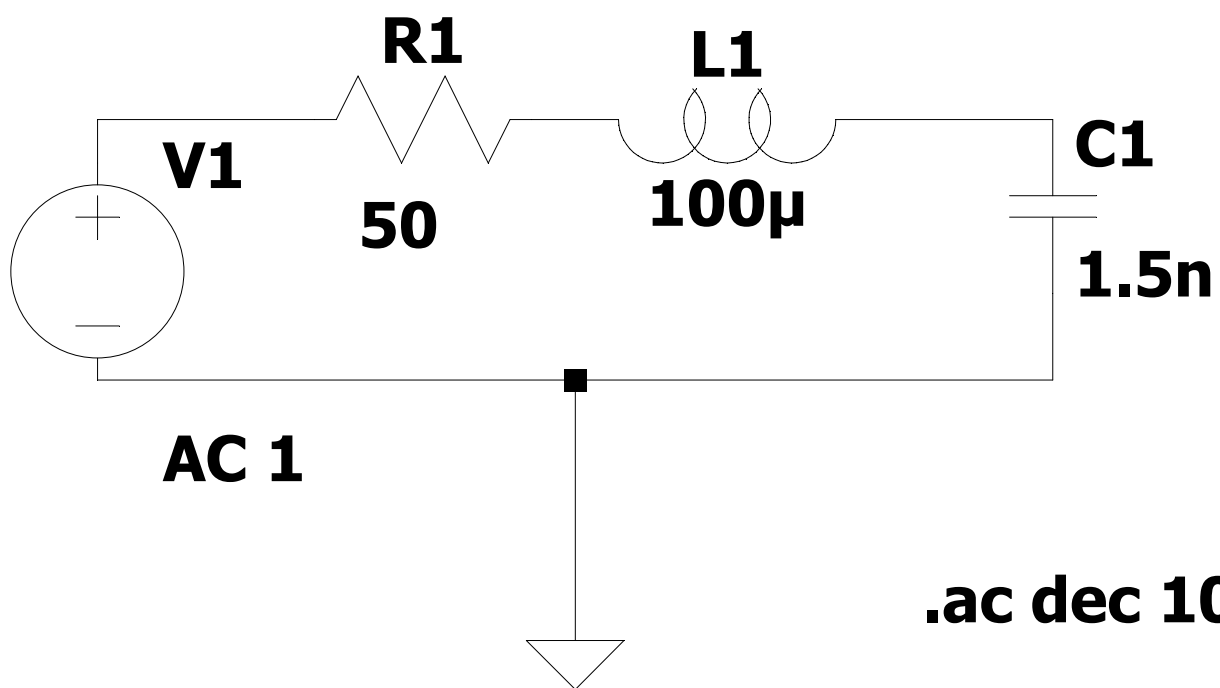

**.ac dec 100 1 1e1**

Supplement: Supplementary file 9 — Source Data [file 41467_2022_29405_MOESM9_ESM.zip › Source Data/Source Code/Fig1C_LtSpice_Simulation_Files/Circuit_Diagrams/LCR_Circuit.pdf]

**.ac dec 100 1 1e12**

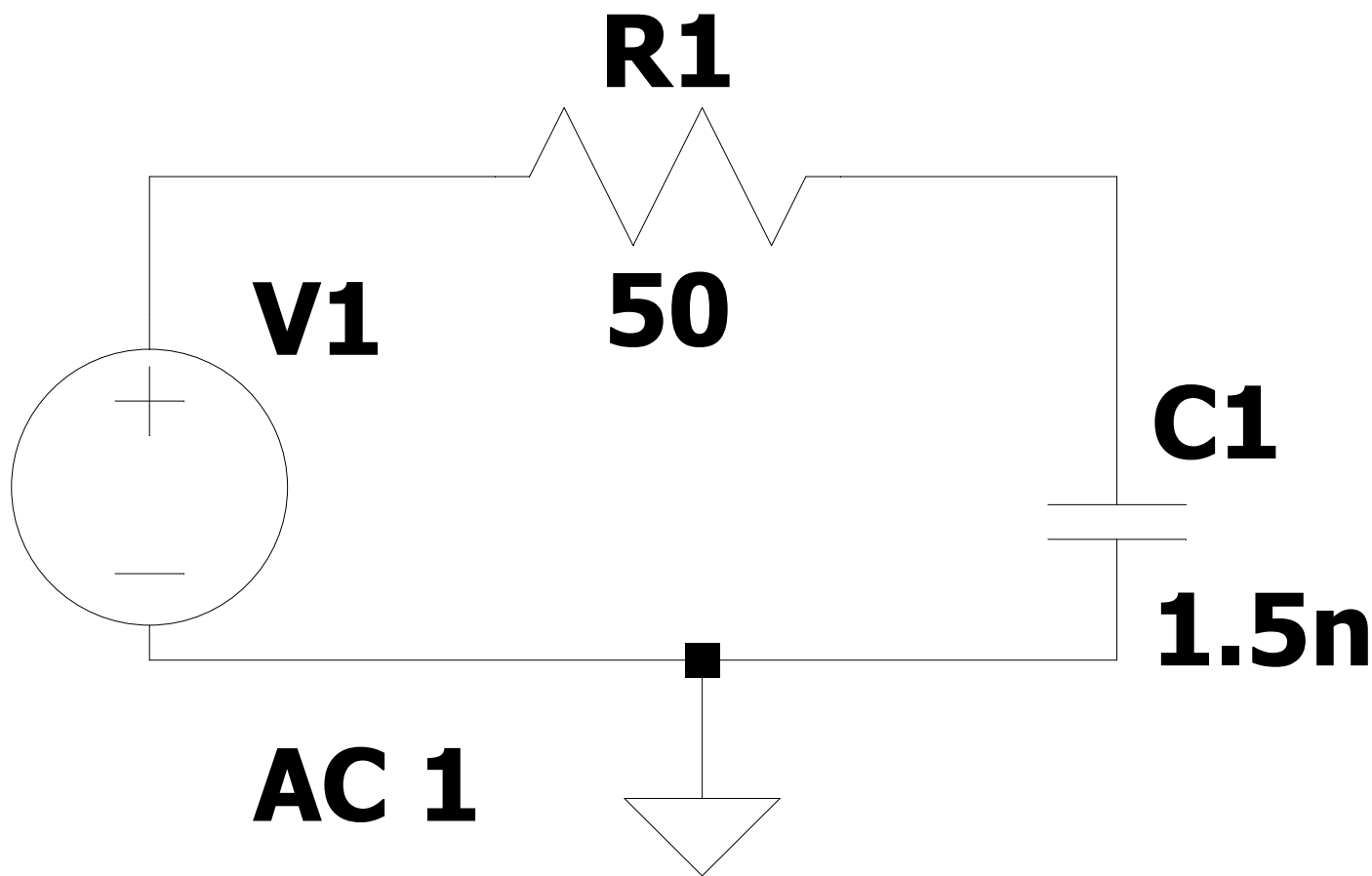

Supplement: Supplementary file 9 — Source Data [file 41467_2022_29405_MOESM9_ESM.zip › Source Data/Source Code/Fig1C_LtSpice_Simulation_Files/Circuit_Diagrams/RC_Circuit.pdf]

```
.step param R list 500 700 900                                .ac dec 100 1 1e12
```

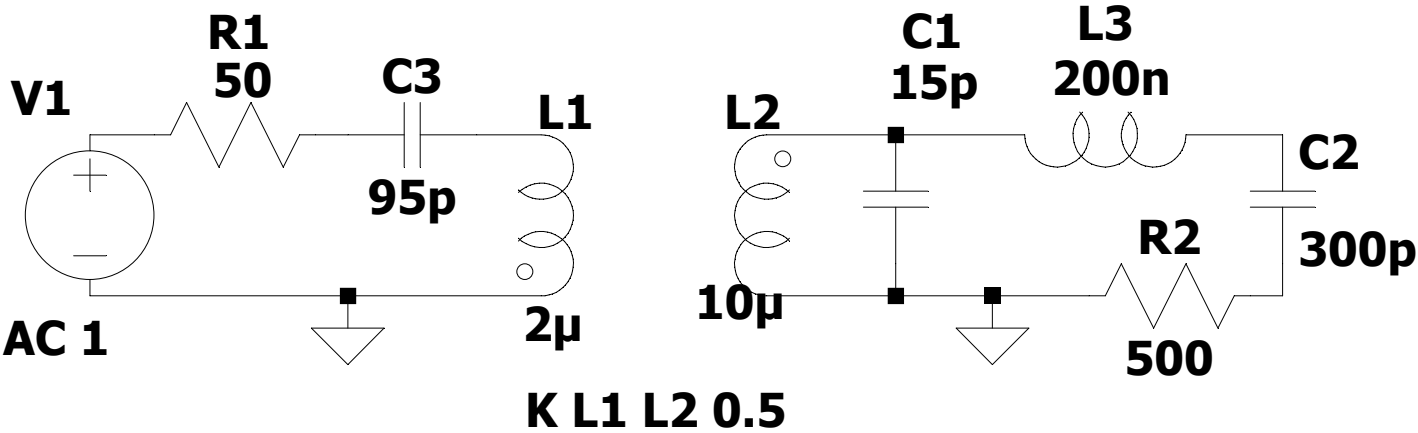

Supplement: Supplementary file 9 — Source Data [file 41467_2022_29405_MOESM9_ESM.zip › Source Data/Source Code/Fig1C_LtSpice_Simulation_Files/Circuit_Diagrams/Smartphone_Circuit.pdf]
